# Pinel: A Biographical Study

**Published:** 1860-04-01

**Authors:** Casimir Pinel


### Art. V.—PINEL: A BIOGRAPHICAL STUDY.

By his Nephew, Dr. CASIMIR PINEL.

(*Translated from the French.*)

Pinel* from his earliest years evinced signs of precocious intelligence, and astonished his masters by his aptitude, carrying away all the prizes for which he competed. He received his preliminary education at the college of Lavaur, and the town soon became acquainted with the brilliant capacities of the young Philippe. Castellane, the Bishop of the diocese, used all his efforts to induce him to adopt the ecclesiastical profession. He did so, and putting on the cassock, studied theology with a zeal inspired by a loving and fervent piety. He devoured, so to speak, the fathers of the Church, and drew from their works, from the sacred writings, and above all from the Gospel, those true principles of Christianity from which, alas! so many deviate. He acquired in a few years a very extensive acquaintance with these subjects, and he was able in his turn to teach his fellow-students, who thus became at the same time his pupils.

Pinel frequently joined his family circle† from which he was only separated by a distance of fifteen kilometres; he walked this distance with great rapidity; his constitution was strong, robust, and vigorous; he was short in stature, but his figure was well proportioned; his head was well developed, his forehead large, high, uncovered, and prominent; he had black hair, piercing eyes, an aquiline nose, a rounded chin, and a small mouth; his smile was kind and affable; his physiognomy, which was stamped with benevolence and goodness, exhibited in early life character, reflection, and maturity; his whole personal appearance, his manners, his language, his deportment, his reserved and austere behaviour, inspired submission and respect; he was never treated with familiarity by any of his four brothers or his sister. Though full of kindness, affection, and solicitude, he awed them

* Born April 20th, 1745.

† At the town of Saint-Paul-Capdejoux, arrondissement of Lavaur.

by his gravity and a tone somewhat magisterial, but at the same time always indulgent. During the day, he assembled them for lessons, and before retiring to rest he presided at family prayer, which he conducted with grave attention. He was scarcely twelve years old when he lost his mother,* whose death caused him the deepest grief.

He followed the chase occasionally with his father,† who was a skilful sportsman, but his sensitive soul was pained at the sight of the game which he wounded or killed, and which he regarded afterwards with regret: accordingly the chase soon became but a means of abandoning himself the more easily to his favourite pursuit, namely, study. Taking his gun for appearance sake, he would at one time plunge into the tangled and solitary woods of the neighbourhood, at others he would ramble about the fertile and beautiful meadows of the Agout, or gaining the top of one of the adjacent hills which form a natural amphitheatre, planted with all kinds of fruit trees, vines, mulberry trees, and maize, he would contemplate with admiration the ravishing prospect of the valley stretching at his feet, and to the east at a distance of forty kilometres the chain of mountains which unites the Pyrenees to the Alps. But if Pinel took a gun, he also took books, and when he arrived at a retired spot he would lay down his weapon, and abandoning himself to study would forget the chase for hours together. His favourite authors were in the first instance Virgil and Horace, and later Cicero, Pliny, and Tacitus,—works which he continued to read with pleasure nearly eighty years after.

About the age of twenty-two, and not twenty-seven, as stated by Cuvier, he quitted Lavaur, and renounced the idea of entering the Church. He then repaired to Toulouse, where he studied mathematics with intense ardour. In this science, which he was soon able to teach, he made great progress. My father has often related to me, how that lodging with him in a very modest apartment, he was witness of his zeal for study. Very frequently on awaking in the morning he found my uncle where he left him the evening before on retiring to rest, that is to say, meditating with his elbows on the table and his hands supporting his head.

At this time he delivered lectures in philosophy and sustained a thesis on this branch of knowledge, of which the title sufficiently reveals the serious predilections of his mind. It was "On the Exactitude which the Study of Mathematics gives to the Judgment in its Application to the Sciences." (*De la Rectitude que l'Etude de Mathematiques imprime au jugement dans son Application aux Sciences.*) It is reported that about the same period he was a competitor at the floral games, and was crowned.

In a short time he entered a rich and honourable family, and

* A very pious woman. † A physician and surgeon.

undertook the education of two of the sons, of whom one afterwards followed the career of the magistracy and the other that of political finance. At the same time he walked the hospitals and attended the course of the School of Medicine at Toulouse. He there passed a brilliant examination, and took the degree of doctor in the month of December, 1773.

In the beginning of 1774, after residing some years in this town, thirsting for more extensive knowledge, he set out for the celebrated University of Montpellier, which had just previously lost an illustrious member in Boissier de Sauvages, and where the merit of Barthez was commencing to make itself felt.

There, as at Toulouse, he entered a highly respected family, as tutor to the son of the house, who afterwards became an officer of great genius and merit. Cuvier is wrong in stating that he opened an establishment at Montpellier as a means of livelihood. The time which his tutorship left at his disposal was employed in perfecting his knowledge of the ancient languages, in attending courses of medicine, natural history, and chemistry, and in composing for aspirants for the doctorate theses which were models of correct and elegant latinity. He selected from choice questions relating to hygiene, a branch of medicine for which he had great partiality.

In this town Pinel made the acquaintance of a young man of fervid imagination, but restless and impatient temperament, who had attempted various branches of literature, but whose labours had till then been fruitless. Pinel was the means of leading him back to more positive ideas by giving him lessons in mathematics, and inducing him to read and study together with himself the works of Hippocrates, Plutarch, and Montaigne, and to attend the courses of medicine and the cognate sciences. From this period, they formed an attachment to one another which was never weakened by either time or distance. This fellow-student and pupil, subsequently attaining under the Consulate and first Empire the highest dignities and the most eminent scientific honours, was the illustrious Chaptal, Comte de Chanteloup, who ever maintained a grateful recollection of the lessons and counsel of his friend.

At the same epoch, Pinel, who, as I have already stated, took the degree of doctor at Toulouse, attained the same honourable rank at Montpellier. He attached himself to a young English student, and they taught one another the English aud French languages. It is related that leaving Montpellier for Paris about the commencement of 1778, and not 1772, as has been frequently stated in other biographical notices, they were stopped on the road for want of passports, the simplicity with which they travelled being such as to inspire little confidence in the local authorities with whom they came in contact.

They were allowed, however, after some explanations to continue their route. Forty-three years afterwards Pinel's companion, who in the interval had risen to eminence as a physician in his own country, visited France and presented his family to the author of the *Nosographie*.

Pinel carried letters of introduction to Cousin, which he presented on arrival. This great geometer, struck with Pinel's extensive mathematical attainments, was eager to procure him pupils, whose well-remunerated lessons assured him an honourable independence, and put it in his power to give himself to the study of literature, science, and, above all, of medicine.

It happened by chance that in the same house with Pinel there resided a very diligent young student towards whom he experienced an active sympathy, which the latter frankly returned; a strict and lasting friendship was the result, and this friend of my uncle was the excellent Desfontaines, who afterwards became Professor of Botany at the Jardin des Plantes and Member of the Institute.

In a short time Pinel's position became still more independent, and he was able to dispense with the income derived from his mathematical pupils; from this time he attended the hospitals more assiduously, resorted frequently to the libraries and academies, translated for the press, wrote for several scientific periodicals, particularly the *Journal de Paris*, in which he published numerous articles on medicine, physics, and philosophy, obtaining at the same time a small but select medical practice. Towards the close of 1782, he obtained the editorship of the *Gazette de Santé*, in which he published a part of his treatise on hygiene. In 1784 he translated from the English, Cullen's "Institutions of Medicine." For this translation he was paid, as he informs us, 1000 francs. It appeared in 1785, and not 1784, as elsewhere stated.

In the course of his philosophical and mathematical studies, Pinel had naturally been led to consider the works of Borelli, which he greatly admired. It is well known that the Neapolitan physician displayed great talent, often successfully, in endeavouring to apply the laws of statics and mechanics to physiology. The French physician composed two memoirs, the one relating to partial motions of the extremities, which he read before the Royal Society of Montpellier in 1777; the other to motions of the entire body, which he intended to have read before the Academy of Sciences at Paris. They formed a portion of the articles on surgery, comparative anatomy, and zoology, which he published in the periodicals before alluded to. In 1785 and 1786 he communicated to the Academy of Sciences memoirs on various dislocations, extracts of which were reproduced in the scientific journals of the day. In 1786 he published in the *Journal de Physique* an article on original defects of the genital organs, and the apparent or real characteristics of hermaphrodites, founded on a case which came under his own notice. He subsequently wrote for the same journal three other memoirs: the first on the structural formation of the elephant's head; the second on the retractility of the claws of certain carnivorous animals; and the third on the mode of preparing the skins of quadrupeds and birds. In 1788 he edited a new edition of Baglivi with notes and commentaries. In 1789-91 an abridgment of the "Philosophical Transactions" was published, and Pinel out of fourteen volumes translated three himself—one on chemistry, another on anatomy, and a third on medicine and surgery. He also worked upon an additional volume devoted to materia medica and pharmacy, About 1793 he made an interesting note on an ossification of the brain, which was exhibited at the Academy of Sciences in 1753 by Baron, and which had been preserved by Deyeux.

In 1783 the melancholy loss of a friend, who fell into a state of mania from an excessive enthusiasm for glory, coupled with insufficient means to contest it, directed his attention to the study of mental alienation, and as he himself afterwards stated in his "Memoir on the Moral Treatment of Insanity" (*Mémoire sur le Traitement Moral de la Folie*), and in his "Medico-Philosophical Treatise" (*Traité medico-philosophique*), his admiration for the judicious precepts of the ancients with regard to this malady was increased; and from this period he *commenced a series of observations in a lunatic asylum (Maison de Santé Belhomme), which he pursued during five years, on mania and the application of moral remedies to the disease.* At this period Pinel commenced the publication in the *Gazette de Santé* of articles relating to nervous and mental disorders. In 1788 he communicated to the Royal Society of Medicine a work on the distinctive features of the various kinds of mania and the modes of treatment, which was the result of his observations at Belhomme and of his private practice: in 1789, he published in the same Journal a note entitled, "Observations on the moral regimen best calculated to restore the wandering reason in certain cases of mania" (*Observations sur le régime moral qui est le plus propre à rétablir, dans certains cas, la raison égarée des maniaques*). He also wrote for the *Journal Gratuit de Santé* in 1790 a memoir full of interest, entitled, "Medical Reflections on the Monastic Life" (*Reflexions Médicales sur l'Etat Monastique*), where he related a remarkable case of erotic melancholy cured by garden labour and baths. In the early months of 1791 he inserted in Fourcroy's

Journal, called *La Médecine éclairée par les Sciences Physiques*, several articles on melancholic suicide; on the 30th August, 1791, the Royal Society of Medicine offered a prize for an essay "*On the most efficacious means of treating invalids who become insane before old age.*" Pinel sent in a memoir which received honourable mention on the 28th August, 1792, and which caused the judges charged with its examination, of whom Thouret was one, to form a high opinion of the author; to this work was appended the motto from Celsus, "Gerere se pro cujusque natura necessarium."

Cabanis, Cousin, and Thouret, friends of Pinel, were about this time placed at the head of the Board charged with the administration of the public hospitals, some of which and amongst others Bicêtre, were then in a deplorable state. They were well acquainted with his merit and the attention he had already paid to the subject of insanity; they thought he was the only man in France, as Pariset says, capable of remedying the evils and disorders which prevailed in the lunacy department; they therefore hastened to appoint him chief physician of that hospital.

It was during the troubles of the Revolution, a memorable but sanguinary epoch of our history, that this appointment was made. Biographers are not quite agreed as to the precise period. Some say it was in the commencement, others that it was towards the close of the year 1792. From several passages in the *Traité Medico-Philosophique* one might infer that Pinel was already at Bicêtre in 1792: thus he gives an account of the massacres of the month of September, and relates a highly dramatic episode relating to this subject. It appears evident from what he says that he was on the spot at this time. In another part of the same book he expresses himself thus: "My observations were resumed at Bicêtre on my nomination during the first year of the Revolution." By *Revolution* should be understood the Republic. In the *Nosographie* he says: "My appointment to the post of Chief Physician of the Infirmary of Bicêtre about the first year of the Republic." Again, in another place he writes: "The Hospital of Bicêtre confided to my charge, under the title of Chief Physician, during the years II. and III. of the Republican era, opened out to me a clear stage on which to follow out those *researches commenced at Paris some years previously.*" The letters in my possession written in February, July, and November, 1792, and in January, 1793, contain no reference to his sojourn at Bicêtre; on the other hand, the register of the hospital fixes the 25th August, 1793, as the date of his appointment, and that of his installation in the early part of September. This date agrees with the close of the first year of the Republic. After this it must be taken that it was in the latter end of 1793, and not 1792, that Pinel presented himself at the Hôtel de Ville in order to obtain authority from the Commune to loose the fetters of the lunatics at Bicêtre, since Couthon, who presided over this assembly, had retired from the scene of politics in the latter half of the year 1792, and did not make his re-appearance until January, 1793; besides, it was probably the revolutionary tribunal appointed on the 10th August of this year which is here in question.

It is of little importance, however, whether Pinel was appointed in 1792 or in 1793; that which it concerns us to know and to remember, and which constitutes one of his greatest claims before posterity, is that a short time after his installation at Bicêtre he partially realized those ideas of reform which he had conceived, and already applied on a smaller scale, and that by so doing he radically altered the lot of the insane. This reform it is—as great philosophically considered as medically—which, continued and consummated by Esquirol and M. Ferrus, has wholly transformed the asylums for the reception of lunatics.

In our days we can scarcely form any idea of the then deplorable condition of these houses. If we picture to ourselves low, damp, and infected dungeons, without light or air, fitly designated by the name of cells, containing a wretched stump-bed, or a rotten straw mattress laid on the stone floor; if we imagine human beings, naked or covered with rags, nearly always furious, chained, and shut up in these places of desolation and misery, real tombs which they never left but for their last resting-place; if we conceive ferocious keepers chosen from amongst convicts, treating these beings as brute beasts, making use of the most barbarous expedients, overwhelming them with injuries, mocking them with insulting jibes, mercilessly beating or waging with them terrible and often sanguinary struggles, throwing down before them disgusting and insufficient nourishment, leaving them without water even to quench their thirst, or clothing to protect them from the cold of winter, and exposing them in this sad condition for the amusement of curious visitors; if we imagine, I say, these unhappy beings believed to be incurable, abandoned by their families, deprived of medical care, pale, ghastly, and haggard, stagnating in their own dejections, groaning under the weight of irons which lacerated their limbs, emaciated by repeated blood-letting, excited by their horrible sufferings, and the inhuman treatment to which they were subjected, we shall then have a very incomplete idea of the frightful state of lunatic asylums in general, and of Bicêtre in particular, at this epoch.

Pinel's compassionate heart was grieved by the spectacle, and the aspect of the unhappy patients, in some of whom perhaps the light of reason was not altogether lost without hope of recovery. Like a new Messiah he undertook the work of regeneration and deliverance to which Providence had called him: in him kindness and gentleness, pity and humanity, justice and philanthropy, philosophy and science, made their entry into this habitation of misfortune and despair.

Pinel found Pussin, an uneducated but trustworthy man, superintendent at Bicêtre, who understood his ideas, and seconded his views of reform. He forgot, as he says, that a doctor's cap had never adorned Pussin's head, and he had numerous interviews with this subordinate, in order to become acquainted with the antecedents of his patients, and frequent conversations with those of them who were able to understand him; he studied their character, soothed the self-love of some, promised to satisfy their reasonable requests, combated with complacency and kindness, or when needed with firmness, the delirious ideas of others, endeavoured to secure the confidence of all. He gave them hopes of an ameliorated lot, and of even returning to their families if they would but follow his advice and not give themselves up to their extravagant and disordered notions. When he felt certain that his influence and ascendancy over them was established on a sufficient basis, he undertook to relieve them of their chains, and to allow them to leave their sombre and unhealthy habitations; granting them in the first instance a limited degree of liberty within the precincts of their respective divisions. The Government of that day was jealous, and Pinel was watched and suspected. It was therefore with difficulty, and only after repeated requests, that he obtained authority to strike off the fetters of the insane. So distrustful were the rulers of France at this period that they saw, even in the liberation of lunatics, the slaves of ignorance and barbarity, an act which might be favourable to aristocracy, and dangerous to Republican institutions.

The celebrated Couthon, who presided over the formidable Commune of Paris when Pinel applied for this authority, went over to Bicêtre the next day, in order to satisfy himself that the request concealed no project inimical to the democratic government. When he saw the lunatics whom Pinel wished to release from their fetters, he turned to him and said, "Are you mad yourself, that you wish to set at liberty these ferocious beasts?" "No," replied Pinel, with simplicity and firmness, "for I am certain that these unfortunates are only thus violent and so extravagant because they are chained. I am convinced that when they are so no longer they will compose themselves, and may, perhaps, again become reasonable." "Do as you like," was Couthon's answer as he left.

From this moment Pinel set to work, and the next day he removed the chains from fifty lunatics, and from thirty more some days after. It is this scene, so beautiful and admirable for its science and humanity, which does so much honour to Pinel, and of which his family are so justly proud, that the Academy of Medicine has sought to commemorate by a picture which ornaments the hall in which their meetings are held.

In those days, however, the most praiseworthy actions were often misinterpreted, and malevolence, ignorance, or political fanaticism sometimes exposed them in an odious light. It was spread abroad that Pinel had released the lunatics from their fetters with bad intentions, and under this pretext, a furious mob one day brought him *à la lanterne.* Chevingé, an old soldier of the French guards, rescued him out of their hands, and thus saved his life. This man was one of those lunatics liberated by Pinel, afterwards cured, and ultimately taken into his service.

The reforms wrought by Pinel from a medical and philanthropic point of view, excited immense interest in all civilized countries, and from that time they have gradually spread themselves amidst the applause of generous and enlightened minds. At the same time it must not be forgotten that the ideas pervading all minds were in favour of amelioration, that the philosophy of the eighteenth century tended to bring them to light, that they were entertained even by the authorities of the day, and that for ten years opportunity had been sought to introduce them into lunatic asylums.

Attempts of this kind had been partially made in various countries, and some physicians, particularly in the hospitals under their care, had endeavoured to change the abominable system then pursued towards the insane. Thus, the patients belonging to the hospital at Saragossa, in Spain, had for long enjoyed a certain amount of liberty, and manual labour was also employed there with advantage. This asylum, which was open for the reception of patients of all countries and all religions, bore as its motto, *Urbis et Orbis.* In some asylums also in Holland, Belgium, Savoy, England, and France, efforts were made to mitigate the lot of this unhappy class. But what were all these imperfect and timid attempts, these incomplete schemes, this return towards the precepts of the ancients, which Pinel had extolled and put in practice ten years before his appointment to Bicêtre, as compared with the radical reform based on the most rational principles of medicine and philosophy, stamped with the true spirit of Christianity, to which a grateful posterity has attached the name of the French Psycopathist?

After researches extending over a period of four years, both at Bicêtre and the Salpêtrière, of which he had become chief physician, Pinel published his conclusions in the *Mémoires de la Société Medicale d'Emulation* for the years V. and VI. These works, together with those he had written before his appointment to Bicêtre, formed the groundwork of the first edition of his "Medico-Philosophical Treatise upon Insanity" (*Traité Medico-Philosophique sur l'Alienation Mentale*) in the year IX (1800). He subsequently completed them by further observations and more extended applications in a second edition published in 1809.

It is impossible to contest the originality, the high merit, and the great importance of this didactic work, which has rendered immense service in popularizing Pinel's doctrines. When this Treatise appeared, there were no works in mental science serviceable as guides in such matters. The few English books printed in the last quarter of the eighteenth century, such as those of Arnold, Crichton, Ferriar, Haslam, Perfect, &c., possessed but a moderate degree of interest, presented vague and insufficient precepts, and contained no really elevated medical views capable of directing the physician in the deplorably chaotic state of the lunatic asylums of the day. Those which had been edited in Germany and Italy, without excepting even the works of Locher, of Greding, of Langermann, of Chiarugi, possess no greater merit. Dr. Daquin published at Chambery about the middle of 1791, and again in 1804, a work entitled *De la Philosophie de la Folie*, which has remained almost unknown, and is not even mentioned by the majority of psycopathists and biographers. This book, although full of generous and philanthropic sentiment and commendable on many accounts, is, nevertheless, destitute of learning, science, and any special ideas; it is written in an incorrect style, and contains a host of erroneous propositions, such as those relating to suicide, the curability of insanity, the venereal desires of the insane, the influence of the moon, &c. His classification of mental disorders will not bear the slightest examination. In the second edition, dedicated to Pinel, he did not profit by the progress made in medical science; he limited himself to the praise and exaltation of the *Traité Medico-Philosophique*. The following is an extract from the dedication :—

"I addressed the first edition of this work to Humanity, because the subject appeared to make it a duty that I should do so; but to-day I fulfil one, to me much more satisfactory, and much more appropriate to the matter, in dedicating this second edition to you, because I see in you that rare virtue personified. Your work on mania displays at once the generous sentiments of a refined mind, and the fertility of genius. We find in it a sympathizing feeling for the woes of others, beside the salutary resources of the art of relieving them."

No one, and Daquin less than any, ever contested with Pinel the honour which he acquired by the reforms made at Bicêtre; for some years, however, attempts have been made not exactly to tarnish its glory, that would be difficult, but to lessen it by sharing it with Daquin, on the pretence that to the latter belongs the priority of reform in the treatment of the insane. I believe that I have in another place (see my note on the reform in the treatment of lunatics, 1854, and my letters in the *Journal des Connaissances Médicales* of Dr. Caffe, 1858,) shown the real value of these pretensions, and that Daquin's medical fellow-citizen who raised them, and the two other gentlemen who supported his opinion, had not sufficiently studied the question, and were not acquainted with Pinel's labours. They made the mistake of confounding two different and distinct things: the priority of ideas on the moral treatment which Pinel never ceased in his writings to trace back to the ancients, Aretæus, Celsus, and Cælius Aurelianus; and the application and realization of these ideas, which could only be accomplished with advantage on a wide field, such as Bicêtre or the Salpêtrière, whence they might spread their salutary influence through the whole world.

On a mere comparison of the dates of publication of the *Traité de la Philosophie de la Folie* and the *Traité Medico-Philosophique*, and without taking account of anything Pinel had done before his appointment to Bicêtre, it has been contended that Daquin preceded Pinel in the reformed treatment of the insane; that he enunciated novel ideas; that they were new and bold, considering the time when they were promulgated; that he ought to be regarded as the inventor of the moral treatment; that his ideas were the fertile germ of the great reforms accomplished by Pinel, Esquirol, Conolly, and Leuret; that he, first of all, indicated and attempted that system to which Pinel attached his name. It is impossible that any one who has taken the trouble to study the history of medical psychology, and who knows anything of the labours of Pinel, should admit such propositions, after an attentive perusal of Daquin's book. In fact, this author has enunciated no new idea, and one might quote from other writers a host of passages in which what he has written is more formally and more scientifically expressed. It may be seen even that his philanthropical notions are but the pale reflection and a repetition of what had been said before. He inaugurated no reform, he did not remove the chains, for he does not even suggest such a thing, and at his death, twenty-five years after he wrote, the lunatics at Chambery were in the state in which he found them. None of the improvements which he proposed, or of the desires and hopes which he expressed, had been either attempted or realized.

Daquin limited himself to the permission granted to a few of his patients to walk individually in an enclosure or orchard belonging to the asylum, where he acknowledged that it was impossible to leave them because they destroyed the fruit. Their lot moved him with pity, he procured them some alleviations, and was sorry he could do no more, nevertheless it is wished to cite his good intentions, emanating from a generous but powerless disposition, his abortive and unproductive attempts, as an innovation and even the inauguration of reform. I ask every impartial man what there is in common between these, Daquin's imperfect and all but unknown acts, and those of Pinel, so fertile in the happiest results.

I feel it my duty to say one word as to Pinel's silence with respect to Daquin, upon which stress has been laid by those who are unable to reply to the objections founded upon facts and dates. They have gone so far as to pretend that he acted under the influence of rivalry and jealousy. Such insinuations cannot affect Pinel, whose morality and scientific probity are above these retrospective attacks directed against his memory, and if they should create astonishment it would be because they proceed from the pens of honourable men who, in exhuming Daquin's unknown work, have attempted to make him a reputation at Pinel's expense.

It seems to me that Pinel's silence may be easily accounted for, and one would naturally suppose either that the *Traité de la Philosophie de la Folie* was unknown to him; or, as appears to me most probable, he had forgotten it in the midst of his numerous occupations.

However this may be, I think he was perfectly right to maintain silence, for the flattering compliments of Daquin did not oblige him to speak his mind upon the book, in which there was much to criticise. The second edition was but a paraphrase of the first, and in no way on a level with the state of science. The author had not profited by the eminent labours of the French psychologist, and the latter therefore had great reason to complain.

France had produced no work treating *ex professo* of insanity. In the last twenty years of the century there appeared—1st, a document full of interest, edited under orders of the Government, by Colombier and Doublet (1785); 2nd, some Ideas by Tenon (1788); 3rd, a work by Iberti, on the Lunatic Asylum at Saragossa; 4th, a Report read before the National Assembly by Larochefoucauld (1791); 5th, some Thoughts by Cabanis (1793); 6th, an account of the Lunatic Asylum at Amsterdam, by Thouin (1796); 7th, some Letters in the *Bibliothèque Britannique*, on a New Establishment for the Cure of the Insane, by Dr. Larive (1798); and lastly, the Articles and Memoirs of Pinel, of which I have spoken (1784—1798).

It was under these circumstances, then, that the *Traité Medico-Philosophique sur l'Alienation Mentale* was published.

The grand plan of the work which Pinel proposed to himself, and which he so worthily accomplished, may be shortly summed up as follows:—To give an historical sketch on the subject of mental disorders; to discuss the value of the works which treat of them; to classify these disorders under four principal divisions, of which one, Melancholy, presents itself under two different forms depending upon moral oppression or expansion; to submit a selection of observations; to throw light on the most frequent causes; to explain the great importance of the original disposition of the different passions, &c., in their etiological relations, and the utility of psychological knowledge; to determine the somatic and psychical characters of each genus; to describe and make known, in the first place, that species of mental alienation consisting rather in the perversion of the affective faculties, the instincts, the desires, of the moral sense, than in the lesion of the intellectual faculties, which may be or may appear to be intact; to develop the medico-legal doctrines which proceed from the existence of those sometimes transitory exhibitions of mania without delirium, moral insanity and instinctive monomania; to demonstrate to the judges that numbers of persons brought before them as culpable, and convicted as criminals, are but madmen; to snatch unhappy sufferers from the scaffold or the galley, thus opening out to modern psychologists a new career which they were destined to run, with advantage to justice and humanity; to indicate general rules for the distribution of asylums; to establish general precepts that ought to be followed in the physical and moral treatment of the insane; to make apparent the utility and necessity for manual labour and isolation; to reprobate with energy the use of all violent means; to draw up statistical tables; to undermine ancient prejudices by force of logic and science; to overturn the worm-eaten edifice erected by ignorance, barbarity, and inhumanity; and not contenting himself with merely giving precepts and advice, to apply and realize them at the expense sometimes of his health, his repose, and his liberty; and finally to culminate in general considerations, abounding with philosophical and medical ideas of the deepest interest.

I admit that Pinel's classification was defective, and that it required modifications already made, partly by Esquirol and M. Ferrus, his pupils and his learned and worthy followers; but it is nevertheless the basis of all those which have been proposed up to this day.

Granting that Pinel has at times confounded dementia and idiocy; that he has classed mania accompanied with partial delirium under the head of mania without delirium; that with respect to some forms and species of madness, the seat of disease, and pathological anatomy, his work leaves somewhat to be desired, it must still be acknowledged that he has traced with a sure, vigorous, and practised hand, all that relates to the moral and physical *régime,* and that little has since been added upon this point.

Pinel's book is more than a mere medical work; it is full of philosophical views, and of moral precepts and doctrines which may be as useful to the physician as to those whose occupation concerns psychology, education, legislation, justice, and political administration. Pariset, indeed, has not thought it too much to say that it ought to be the manual of physicians and rulers.

Pinel's system of moral therapeutics is not merely based on the most enlightened science and the most consummate experience, but also on the purest virtues, the truest and noblest sentiments, such as justice, kindness, goodness, and charity. It may also be said that it will ever be in all places and in all time, as it is to-day, that part of mental medicine which is the most true, the most positive, and the least contestable.

The first edition of the "Philosophical Nosography" (*Nosographie Philosophique*) appeared in 1798, and the sixth was published in 1818.

I have no need to state here, for every physician is aware of the immense medical revolution caused by the appearance of the *Nosographie,* which for twenty years was the sole guide both of students and physicians, as well in France as in foreign countries; I will only say that this work forms the crowning point of the author's reputation.

Dupuytren has erroneously stated that the *Nosographie* gained the decennial prize; it appears from what Pariset has written, that Hallé, who was the referee named by the commission issued on this subject, declined to pronounce between the competitors, Corvisart and Pinel, who were both his friends. But it is easy to see, from his remarkable report, that the comparison was altogether in favour of Pinel. Could it be otherwise? The one work was limited to a description of the disorders of the organs of circulation; the other embraced the whole subject of internal pathology. On the one side was a study of narrow extent, restricted research, and limited observation on the maladies of the heart and great vessels: on the other, an attempt to establish a new doctrine, to create a new classification, to examine important works, to effect a philosophical analysis of a subject involved in labyrinthine intricacy; to give order, method, and simplicity to a science which numerous and different theories and systems had rendered more and more obscure, unintelligible, and uncertain; above all, to combat the exclusive pretensions of solidism and humorism; to recall the science of medicine, by incessant efforts, to the true principles traced by Hippocrates. Such, in a few words, is the difference between the works of Corvisart and Pinel, and which Hallé displayed with so much talent.

Pinel also published a "Treatise upon Clinical Medicine" (*Traité sur la Médecine Clinique*), which went through three editions between 1802 and 1815; and he wrote for the *Encyclopédie Méthodique*, for the *Dictionnaire des Sciences Medicales*, and for Transactions of the Institute. It should not be forgotten that it is to these reflections or to the observations made by Pinel upon Inflammation, in 1791, that the "Treatise on Membranes" (*Traité des Membranes*) owes its birth, and that a spark of his genius, as Dupuytren says, lighted that of Bichat.

On the formation of the *Ecole de Santé*, in 1794, Pinel was called to the chair of Hygiene conjointly with Hallé, and afterwards to that of Internal Pathology, on the death of Doublet, in 1795, which he continued to hold when the *Faculté de Médecine* was instituted. He was nominated a member of the Institute in 1803, in the Zoological section; Chevalier of the Legion of Honour at the institution of that order; consulting physician to the Emperor in 1805; Chevalier de St. Michael in 1818; honorary member of the Academy of Medicine at its formation; and he was one of the eleven professors who witnessed the dissolution of the *Faculté de Médecine* under the ordinance of 1822.

Pariset and Cuvier have represented Pinel as in most wretched circumstances during the first year of his residence at Paris: this is an error disproved by my uncle's own letters. Cuvier also pretends that he fell into a state of melancholy in consequence of his penury, and that he would have lapsed into despair but for his friend Savary, who revived his courage and procured him some distractions: this story has no more foundation than another he relates, when he says that the only means he had of providing for his necessities was to take a situation in a lunatic asylum. Pinel never resided at the Belhomme Asylum, of which he was merely the physician. It is a well-ascertained fact that on his arrival at Paris he obtained, through Cousin's introduction, as I have already stated, pupils in mathematics, whose fees amply sufficed for his simple and modest wants; his correspondence leaves no room for doubt upon this head.

Pinel was acquainted with nearly all the men of that day who were in any way celebrated either in literature or science, and amongst others with D'Alembert, Condorcet, Hallé, Lavoisier, Berthollet, Labillardière, Daubenton, Savary, Fourcroy, Thouret, Cabanis, Roussel, &c.; and he was intimate with several of them. The two last-named introduced him to the choice and witty circle of Madame Helvétius, who presided over her réunions with a grace and amiability which added greatly to their charms.

Pinel's timidity and modesty were such as often to paralyse his resources, especially before strangers; he also expressed himself with difficulty, and was unable to arrange and classify his numerous ideas with sufficient rapidity, so that he exhibited a kind of embarrassment and reserve which caused him to be ill appreciated by those not familiar with him. It is related that on being presented by his friend, Desfontaines, to Lemonnier, physician to Louis XVI., with a view to his being appointed physician to the King's aunts, he was scarcely able to say a word, and that he stood dumb before the princesses, who formed a very low opinion of his merit, and declined his services.

Pinel competed three times for the chair of *Docteur-régent de la Faculté*, and was each time defeated; about the year 1784, he offered himself again, having as he thought a better chance of success. By accident or ill-luck, it turned out that his competitor was a man he had known at Montpellier, and who there applied to him to prepare his thesis. Quickly perceiving that the man was extremely ignorant, he chose a subject most likely to accord with his aptitude, and composed a thesis for him on equitation, judging that as an old gendarme he might be able to sustain it without much difficulty; the result was that the candidate was received with great applause. Emboldened by this success, he presented himself at Paris to contest the chair of *Docteur-régent*. He was tall of stature, his delivery was fluent and sonorous, his assurance imperturbable, his learning contemptible. Pinel was under the average height, his air was timorous and embarrassed, his voice feeble, his diction toilsome; it need scarcely be added that the sometime gendarme carried the day, and the future author of the *Nosographie* was nowhere. But the vexation of his rejection in favour of a fool full of fatuous pride was easily consoled, and he could afterwards join his friends in their amusement at the tact and appreciation of his judges, demonstrating *algebraically* the chances sometimes afforded by competition to candidates possessing little beyond a tenacious memory and an audacious and vain confidence.

Pinel had a tender and sensitive soul; he loved the grand, the beautiful, and the sublime; he always retained a taste for poetry, which he cultivated in his youth; he was a passionate admirer of the *chefs d'œuvre* of antiquity; he was deeply moved by the perusal and often the mere recollection of certain narratives related by poets or historians, and he identified himself at times with their verse. It is said that walking in the country one day with his friend Savary, the learned traveller, their conversation turned on poetry and love, and the misfortunes they entailed when allowed to overpower the reason. The history of the unfortunate Sappho naturally presented itself to their minds, and in recalling and repeating it to one another, they began to talk of the talents, the poetical enthusiasm, the amorous exaltation, the despair and cruel end of the celebrated Lesbian whom they agreed in considering worthy of a better lot, notwithstanding her excesses. It would appear that the story moved them to tears, and that after a moment's repose they found it necessary to seek distraction in conversing on a less sorrowful subject.

Pinel was a great admirer of Jean-Jacques Rousseau, and Pariset relates that as soon as he arrived at Paris, he hastened to visit, with his friend Chaptal, the tomb of the illustrious philosopher then recently dead. It is asserted that he was so deeply affected by this visit that he passed five days and five nights without sleep; but it is not pretended that he was prevented from giving his lessons as usual.

It was to the house of a Madame Vernet, a relative of the great painter, that Pinel and Boyer conducted and there secreted their friend Condorcet. This excellent woman lived during the revolution at No. 22, in the Rue Servandoni, where she received boarders; she gladly admitted the illustrious outlaw on the recommendation, not of two young gentlemen, as M. Louis Blanc states, but of two serious men of mature age, the one being about thirty-five, the other fifty, whose courageous devotion served, alas! but to prolong for a few months the life of the unfortunate Girondist. I had an opportunity of meeting at my uncle's, when young, Condorcet's widow and also Madame Vernet, who then resided at Verrières. The first was a woman of superior mind, the other was remarkable for her kindly disposition.

With or without reason, physicians in general have the character of being somewhat unbelieving and not very religious. The friends and contemporaries of Cabanis had mostly a reputation for atheism: but Pinel, while highly valuing his friendship and admiring his talent, was far from partaking of all his ideas, and he could say with the man of old—*Amicus Plato, sed magis amica veritas.* He possessed a mind too elevated to permit of his seeing in nature a mere fortuitous arrangement of matter; he believed, on the contrary, with sensible men and the majority of philosophers that a Supreme Being presided and presides over the marvels of the universe as also of our destiny; he sought not to penetrate that which is impenetrable, or to explain that which is inexplicable; he bowed humbly before the infinite, a subject which cannot be discussed without exposing the mind to doubts little likely to console it. Without being an active Catholic, he possessed that religious turn of mind which, favouring the study of the natural sciences, and breathing a morality at once mild, persuasive, and tolerant, is the enemy of fanaticism and superstition, the sublime morality of the ancient philosophy, and, above all, of the Gospel. One day the celebrated astronomer Lalande met Pinel and said to him, "I am preparing a new edition of the Dictionary of Atheists, in which I have devoted an article to you." The latter replied, "And I am about to publish my Treatise on Madness, in which I will reserve a place for you."

Pinel was remarkable for his extreme benevolence and kindness; he was incapable of the slightest jealousy or envy towards his professional brethren, to whom he ever rendered full and entire justice; he may at times have omitted to notice their labours, but he never did so with any blameable intention; his criticisms were always fair and allowable. He never, under any circumstances, replied to his very few opponents, nor did he permit his pupils to do so. Castel and Baumes, more especially the latter, criticised the *Nosographie* with little decency, but he took no offence and made no reply. When his old pupil, the learned author of *La Doctrine Physiologique,* attacked him violently and in terms unworthy of a physician who desired to respect himself, he shrugged his shoulders smilingly and asked if it were possible to maintain a system so exclusive, repelled alike by observation and sound sense. "Let him talk," said he, "time will do justice between us."

Pinel might easily have obtained, like most of his friends, a high political position, either under the republican or the imperial government; but he was too honest and honourable to solicit, much less to intrigue; his love of independence always caused him to reject propositions made to him with this object. His scientific honours were gained solely by his personal merit.

When it was proposed to nominate the chief physician to the Emperor, two men of great learning but of totally different characters were suggested; namely, Corvisart and Pinel. Between these two eminent professors, rivals in glory and science, but always reciprocally regarding and esteeming one another as colleagues, the balance remained for some time undecided. I have reason to know, having heard it at my uncle's from one well informed, that Marshal Lannes, a friend of Corvisart, caused it to incline in his favour.

Pinel might have been promoted to the hospital at Charenton, where the appointment of chief physician was much better remunerated than that which he held: it was offered to him, and he refused it, preferring to remain with the poor patients of the Salpêtrière.

My uncle was very extensively engaged, as consulting physician; papers for advice were addressed to him from all parts of the world, and people came from all countries to seek his counsel. He might have acquired a considerable fortune, and have left great wealth to his heirs; but to this was opposed his beneficence, his generosity, and his extreme confidence, which was often most unworthily abused; his purse was ever open to the unfortunate. But though he was unable to bequeath to his sons great riches, he left them a patrimony of much more worth in a great name and the example of a well-spent life, fraught with virtue and without reproach.

When the dissolution of the *Faculté de Médecine*, and his own dispossession was announced to him, which violated the stability of the professorial office, he made no complaint, and simply asked what provision had been made for instruction. After having heard the names of the new professors, he added, "What will become of medicine?" Some one remarked that he had a right to a retiring pension. "No, no," he replied, "I want nothing; it is my colleague . . . who should have it."

Notwithstanding his modesty, Pinel was not without a certain legitimate pride. I remember that about 1820 he showed me with some satisfaction a letter which he had just received from Russia, and which was addressed, "*Au Docteur Pinel, en France.*"

He was much pleased with his appointment to the position of consulting physician to Napoleon; he writes in one of his letters of 4 Floreal, year XIII.:—

"I have just received a new mark of confidence from the Government, having been nominated one of the consulting physicians to the Emperor before his departure for Italy. This appointment is the more agreeable to me, since it does not involve any active service. My ambition was fulfilled some time ago, and is so now with much greater reason. I am especially pleased that my appointments do not prevent me enjoying a few days' repose from time to time in the seclusion of country life."

In one of his letters also he mentions with pleasure his decoration with the order of Saint Michael, on the occasion of the Duc d'Angoulême's visit to the Salpêtrière.

He related to me also, about the same period, that Napoleon addressed him when he was received at the Institute after his return from Elba, and inquired if lunatics were on the increase. "I replied no; but I thought to myself," said he, with a mischievous smile "that the superior geniuses, the illustrious and ambitious conquerors, were not perhaps without a spark of madness."

In 1802 Pinel purchased a country house between Étampes and Arpajon; he paid down for it, he says, in one of his letters, 60,000 francs. He went to Torfou every Saturday to rest from the fatigues of the week until Monday, or rather that he might study there with greater freedom, where he had a second well-chosen library. It was in this country house, simple but well situated and very agreeable, that he received his friends and disciples. For a long time Mayor of Torfou, he accepted with gratification the homage gladly offered to him at certain periods of the year, particularly at the annual *fête* of the place. The poor of the neighbourhood found in him a benefactor whose counsel was never sought in vain.

Pinel was gifted with an excellent constitution, and a well-poised mind; and thanks to a sober life and freedom from all excesses, he nearly always enjoyed good health. But in 1793 a typhoid fever which he caught in his attendance upon the prisoners at Bicêtre, amongst whom it was very prevalent, brought him to the verge of the grave. He took pleasure in recounting that he mainly owed his restoration to small, often-repeated doses of old Arbois wine, and from a grateful remembrance of this fact, he always had this wine in his cellar, and sometimes on his table.

There was that, says M. Bricheteau, in his manner and appearance which at once set at ease those whom the reputation of the celebrated physician brought to his consulting room; no man was ever more accessible even at the time of his greatest renown and his innumerable engagements.

His countenance was grave, his forehead furrowed with wrinkles, his look as of old was mild, affable, and intellectual. "Looking on him," says Dupuytren, "one might imagine he beheld one of the sages of Greece."

During the latter years of his life he spent some portion of each day in gardening, either in the garden of the Salpêtrière, or at his own country house.

Pinel lost his first wife about 1812, and married again in 1815. His second wife was an excellent lady, who was sincerely attached to him, and bestowed upon him all the care and solicitude which he needed. His family certainly owe to her the prolongation of my uncle's life, and his sons are no less indebted to her for the preservation of the fortune which he had acquired. When the infirmities consequent upon advanced age, and a first slight attack of apoplexy in 1820 led to his retirement from active life, my aunt never more left him for an instant; she lavished upon him every succour and attention, and every proof of affectionate devotion and conjugal love. When the arbitrary ordinance of Corbière was promulgated, by which, when near eighty years of age, he was left with an income insufficient to maintain his household on the modest footing in which he lived, his venerable wife hid from him their straitened position, and did all in her power to prevent his noticing it; she changed none of her husband's habits, but, on the other hand, denied herself in order that she might have it in her power to comply with them. I have myself witnessed this noble conduct, and I am happy to be able to render this homage to the memory of a woman who was devoted and generous not only towards her husband's sons but also towards all his relations.

From 1820 to 1826, Pinel had several other apoplectic attacks which were followed by partial paralysis. The first seizure left but few traces, but those which followed enfeebled and changed his physical organization. During the last two years of his life he resided almost exclusively in the country. A few friends visited him occasionally, and he was glad to see them and very sensible of their kind attentions.

It is generally believed that the last years of Pinel's life were passed in a species of infancy or intellectual weakness; this is an error, as I might prove by the testimony of his intimate friends who visited him to the last. Without possessing all the activity and energy of his formerly brilliant intellect, he nevertheless preserved the integrity of his judgment, the delicacy of his wit, his power of appreciation, and his medical tact; but he was not always able to express his thoughts as he would have wished: he was conscious of this difficulty and of the morbid causes to which it was due. At the same time when he was able to overcome this embarrassment of speech he expressed his ideas with brevity and clearness. He was ordinarily silent, and appeared absorbed in his reflections or incapable of attention; but, on the contrary, he lost nothing of what passed around him. His visitors were therefore at times astonished at the fitness and justice of his laconic and sensible remarks.

M. Ferrus has often told me, and has repeated to me within the last few days facts which he witnessed, and which confirm what I here state. The following is one of the most remarkable. One day during the last year of Pinel's life a young girl who had had a fall was brought to his house in the country, and complained of severe pain in the inferior part of one of the forearms. Some physicians who were present, and amongst others Messieurs Rostan, Ferrus, and Pinel, jun., after having carefully examined the child, were unable to discover any injury; at the same time as motion was very painful and the patient complained of great suffering, my uncle who had not appeared to have taken any interest in the matter, approached and said to his friends, "The child is very young; examine the inferior part of the radius, there is probably a separation of the epiphysis." A new examination immediately proved the justice of Pinel's diagnosis.

On the 15th October, 1826, he returned to Paris in good health, and without anything to denote his approaching end

During the night of the 21st he was taken with violent shivering, which was the prelude of pneumonia, under which he succumbed on the third day, notwithstanding all the attentions of his wife, his sons, and the physicians who had been called in.

An immense crowd accompanied his remains to the cemetery of Père la Chaise, where Dr. Rostan, his old pupil and friend, pronounced with emotion over his tomb a few feeling and eloquent words. Most of the physicians of Paris made it a duty to assist at his funeral, and all the learned bodies of which he was a member sent deputations. It was very touching also to see in the procession a considerable number of old women from the Salpêtrière, who came to pay a last tribute to him who for more than thirty years had been their physician, father, and benefactor.

---

